# Marine Fouling Assemblages on Offshore Gas Platforms in the Southern North Sea: Effects of Depth and Distance from Shore on Biodiversity

**DOI:** 10.1371/journal.pone.0146324

**Published:** 2016-01-08

**Authors:** Tim van der Stap, Joop W. P. Coolen, Han J. Lindeboom

**Affiliations:** 1 IMARES Wageningen UR–Institute for Marine Resource & Ecosystem Studies, P.O. Box 57, 1780 AB, Den Helder, The Netherlands; 2 Chair group Aquatic Ecology and Water Quality Management, Wageningen UR, Wageningen, The Netherlands; Bangor University, UNITED KINGDOM

## Abstract

Offshore platforms are known to act as artificial reefs, though there is on-going debate on whether this effect is beneficial or harmful for the life in the surrounding marine environment. Knowing what species exist on and around the offshore platforms and what environmental variables influence this species assemblage is crucial for a better understanding of the impact of offshore platforms on marine life. Information on this is limited for offshore platforms in the southern North Sea. This study aims to fill this gap in our knowledge and to determine how the composition and the abundance of species assemblages changes with depth and along a distance-from-shore gradient. The species assemblages on five offshore gas platforms in the southern North Sea have been inventoried using Remotely Operated Vehicles inspection footage. A total of 30 taxa were identified. A Generalised Additive Model of the species richness showed a significant non-linear relation with water depth (p = 0.001): from a low richness in shallow waters it increases with depth until 15–20 m, after which richness decreases again. Using PERMANOVA, water depth (p≤0.001), community age (p≤0.001) and the interaction between distance from shore and community age (p≤0.001) showed a significant effect on the species assemblages. Future research should focus on the effect additional environmental variables have on the species assemblages.

## Introduction

Offshore constructions have been known to act as artificial reefs [1–7]. Foundations of wind farms [8–10], oil & gas production platforms [11] and other energy structures [12] add hard substrata to the marine environment, supporting a great diversity of marine life by offering habitat for algae [3,13,14], fish [15–18] and invertebrates [3,17,19–21]. There is on-going debate on whether this effect is beneficial for the life in the surrounding marine environment, and whether or not oil & gas platform foundations should be removed after decommissioning or left in place as artificial reefs, also known as ‘Rigs-to-Reefs’ [22–25]. Knowledge of the effects of these artificial reefs on marine life is of significant importance for understanding the effect of the presence of several thousand offshore energy structures such as wind turbines [8,26] and their subsequent removal at the end of production life [27,28].

Fouling assemblages on offshore platforms have been inventoried in different areas such as the Beibu Gulf in China [6], the Gulf of Mexico [7,29,30], off the Californian coast [15,31–35], the southern Arabian Gulf [36], the Mediterranean [5] and the Celtic Sea [37]. Research has also been conducted on marine fouling on offshore platforms in several areas of the northern and central North Sea [1,3,11,38]. Previous research on the effects of offshore oil & gas structures in the North Sea focussed on marine mammals [39], fish [16,40–43], algae [44,45], corals [11,21,38] and invertebrate assemblages [1–3,40,44,46].

Several abiotic factors have been proposed to explain the species composition of the marine fouling on offshore platforms, such as water temperature [7], platform age [29], depth [3,6,7,47] and distance from shore [48]. The effect of depth on marine fouling has been reported from wind farms [8,49,50] and offshore platforms [4] in the southern North Sea. However, all the available data on invertebrate assemblages on installations in temperate waters were generated in the northern North Sea or from near shore installations that were constructed <10 years before investigation. The southern North Sea has a strong near- to offshore gradient in environmental variables, such as food availability [51], which is absent in the northern parts. Furthermore, large differences in water depth, temperature and salinity exist between the northern and southern parts of the North Sea [52]. Whomersley (2010; [3]) showed that even after 11 years, fouling assemblages still changed in composition. With offshore platforms in the southern North Sea now reaching ages of >40 years, an opportunity presents itself to compare installations of old and young ages and at different locations with different environmental circumstances. This will give insight in the long term effects of proposed developments, such as the short term installation of thousands of offshore wind turbines [26] and is much needed information to aid in evaluating the impact of future Rigs-to-Reefs programmes: to reef or not to reef [27,53–56]?

This study aims to determine how the composition of species assemblages (including epifauna, fish and mobile benthic fauna on and in the visible vicinity of the installation) changes with depth and along a distance-from-shore gradient. The species assemblage on five offshore gas platforms in the southern North Sea was inventoried using inspection footage from Remotely Operated Video robots (ROVs).

## Material and Methods

### Study sites

**Fig 1:**
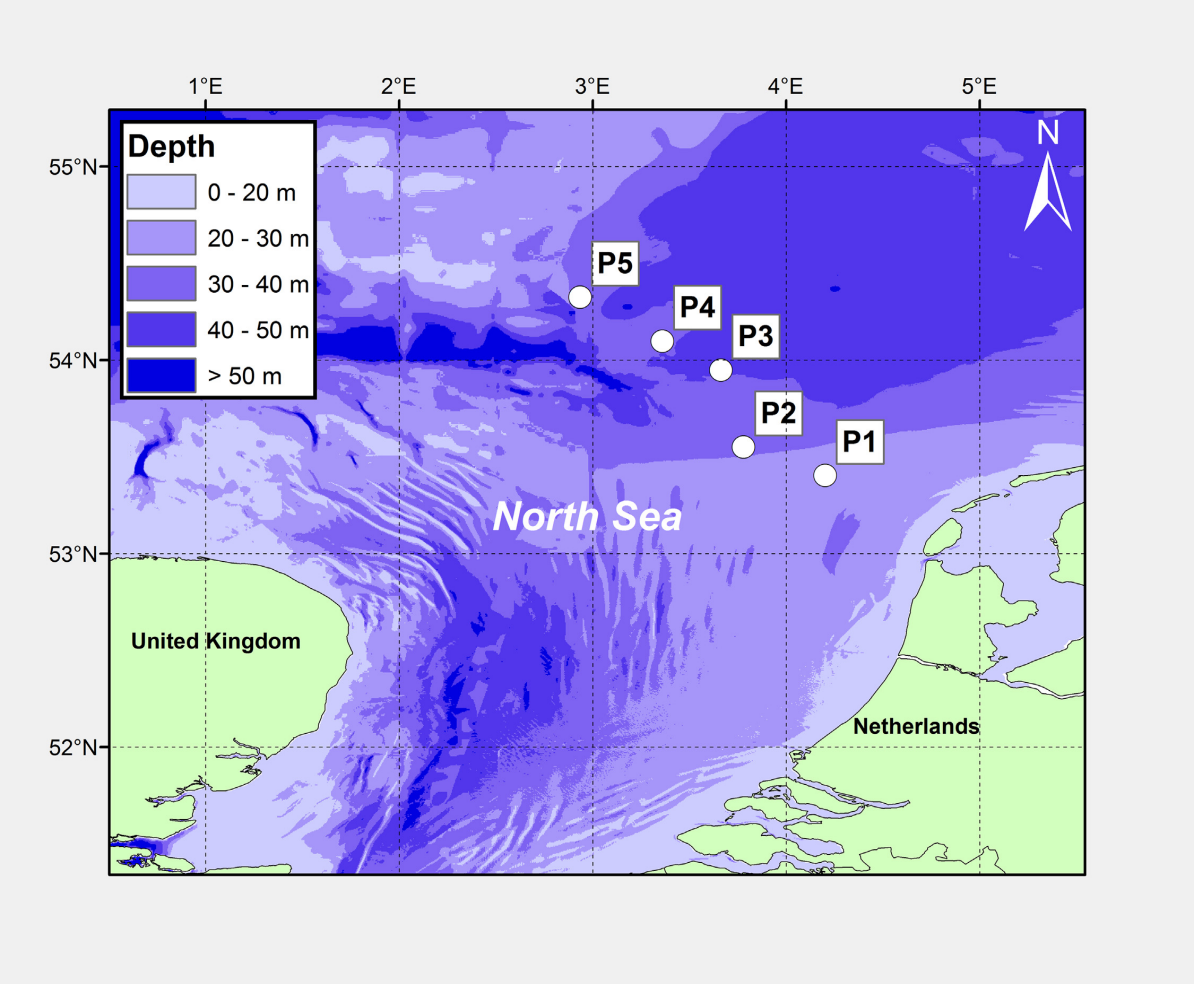
Locations of investigated platforms. Studied locations of five gas platforms in the southern North Sea (○) with bathymetry.

We selected five offshore gas platforms (coded P1 to P5) in the southern North Sea along a gradient of increasing distance from shore, with P1 situated 48 km offshore, and P5 at 177 km offshore (Table 1; Fig 1). The platforms are situated in water depths between 27 and 43 meters, surface water temperatures varying between 4 and 18°C throughout the year [57], on a seafloor composed primarily of sand. The year of structure installation of the structures varies between 1972 and 2009. They are all operated by ENGIE E&P Nederland B.V. and have a steel jacket foundation constructed of 4 to 10 legs with cathodic protection by anodes. Each leg within a jacket provides between ~500 and ~800 m^2^ of surface area available for marine growth, depending on water depth.

**Table 1:** Platform locations, distance from shore, maximum observed depths on the footage, year of installation of the platform, community age (years since last cleaning activities) at the time the video was recorded, for depths < 10 m >.

Platform name (codes)	Coordinates (WGS84)	Distance from shore (km)	Maximum depth of video footage (m)	Year of installation	Community age > 10 m (years)	Community age <10 m (years)
L10-AD (P1)	53°24'12''N, 04°12'03''E	48	27.4	1972	39	5
K9ab-B (P2)	53°33'04''N, 03°46'47''E	80	34.5	1999	12	7
K2b-A (P3)	53°56'55''N, 03°39'44''E	114	42.8	2005	7	7
E17a-A (P4)	54°05'53''N, 03°21'36''E	140	42.5	2009	3	3
D15-A (P5)	54°19'29”N, 02°56'05''E	177	40.0	1999	13	13

### Sampling and analysis

Offshore operators regularly perform technical underwater inspections of the structures, resulting in a large amount of digital video footage, made available to us for analysis. The footage provided consisted of close visual inspection (CVI), showing details of specific parts, e.g. conductors or caissons, and general visual inspection (GVI), giving a complete and systematic overview of each leg of the underwater structure from two approximately perpendicular angles.

For an overview of all species present at each platform, both CVI and GVI footage was used. For the systematic analysis of distance from shore and depth effects, only GVI footage was used. Footage was viewed using VLC mediaplayer version 2.0.5 [58]. To train the observer, all GVI footage was viewed and scored twice during the systematic analysis of species abundance, retaining only data from the second viewing for statistical analysis. Species abundance was estimated from footage while playing, as video stills were often blurry given the low video resolution and fast movements of the camera.

To create replicate samples from identical depths within each platform, all legs from every platform were divided into 5 m depth bands, resulting in a total of 215 unique platform*leg*depth combinations (henceforth named samples). The precise amount of inspected surface area was unknown and we estimate that the area viewed per sample was between 8 and 10 m^2^, assuming that ~50% of the leg was showing in the video and that all legs were the same diameter. Depth and time of recording were visible on the video. To correct for tidal differences, depths were converted to Amsterdam Ordnance Datum using data from Waterbase [57].

Due to the different growth forms, high densities of organisms and limited video resolution, it was not always possible to count individuals for every taxon. Therefore species abundance per sample was assigned a value using an adapted version of the Braun-Blanquet scale, following Leewis *et al* (2000 [59]; Table 2) and Coolen *et al* (2015 [60]). This 1–9 scale allows for a quantitative registration of colonial species and individuals while scaling down bias caused by counting problems from a combination of high densities of individuals with blurry video images. All observed organisms were identified at the lowest taxonomic rank possible. The World Register of Marine Species [61] was used as standard for taxonomical nomenclature.

**Table 2:** Different classes with corresponding analysis value.

Class	Analysis value
1 individual	1
2–5 individuals	2
6–50 individuals	3
>50 individuals, <5% cover	4
5–15% cover	5
16–25% cover	6
26–50% cover	7
51–75% cover	8
76–100% cover	9

Given the low image resolution, blurry video caused by fast camera movement and movement of the organisms, smaller specimens are likely to remain undetected in a sample. Therefore the probability to observe an individual was quantified by defining a detectability score for each taxon. Taxa were scored based on their mobility (1: very mobile, e.g. benthopelagic fish– 5: sessile, e.g. anemones) and individual adult or colonial size (1: small, 3–5 cm– 5: large, >30 cm). By multiplying these scores, taxa were separated in groups of low detectability (scoring 1–6) and high detectability (scoring 7–25). The assumption was made that taxa with high detectability were truly absent when not observed. In high quality footage the size of the smallest organisms or colonies registered was approximately 3 cm, whereas in low quality footage this was approximately 5 cm.

Data on explanatory variables were collected on year of installation of each platform, age of the community at each depth band (the structures are cleaned between 0 and approximately 10 m depth on a regular basis depending on hard marine growth presence), available video length, quality of the footage and distance from the nearest coast. The quality of the footage was scored in consultation between two authors, on a scale of 1 (low quality)– 10 (excellent quality).

Datasets created in this study are publicly accessible through Dryad [62]. For the statistical analyses, R: A language and Environment for Statistical Computing, version 3.0.2. [63] and RStudio version 0.98.994 [64] were used. Taxa with low detectability or with a single observation were removed from the dataset. Species richness (number of species; S) per sample was used to construct a univariate model explaining its relation with the explanatory variables. The collected data were explored following the protocol described by Zuur *et al*. (2010; [65]). To identify outliers, collinearity, relationships and interactions, species richness and all independent variables were plotted using Cleveland dotplots [66], boxplots, pairplots and multi-panel scatterplots (xyplot function in lattice package; [67]). Since non-linear patterns in the relation between species richness and depth were observed, a Generalised Additive Model (GAM; gam function in the mgcv package; [68]) was constructed. Backward selection using Akaike Information Criteria [69] was performed to exclude variables and optimise the GAM. This optimised model was validated by plotting residuals versus fitted values and versus all variables included and excluded during the model selection. PERMANOVA (adonis function in package vegan; [70]) was used to test the significance of the effect depth, community age and distance from shore had on the species assemblages. Also, PERMANOVA was used to examine whether quality of the videos, video length, platform age and community age had a significant effect on the observed species assembly.

## Results

### Species inventory

Approximately 550 minutes of footage were analysed for the five platforms and a total of 30 taxa were identified (Table 3). Nine taxa were observed on all platforms, while four taxa were found on one platform only. After removal of taxa with a single observation or low detectability, 11 out of 30 taxa remained for the statistical analysis.

**Table 3:** All observed taxa (●) per platform with detectability scores (≤6 = low, >6 high).

	Platform					
Taxon	P1	P2	P3	P4	P5	Score
Rhodophyta*	●	●	●	●	●	20
Porifera	●	●	●	●	●	15
Hydrozoa**			●	●	●	5
* Ectopleura larynx*	●	●	●	●	●	5
Tubularia indivisa*	●	●	●		●	-
Anthozoa						
Diadumene cincta*	●				●	-
* Metridium senile*	●	●	●	●	●	20
Sagartia elegans*			●			-
* *Hexacorallia	●	●	●	●	●	15
* Alcyonium digitatum*			●	●	●	20
Annelida						
* *Serpulidae	●	●	●	●	●	5
Arthropoda						
* Cancer pagurus*	●	●	●	●	●	20
* Necora puber*	●	●	●	●	●	12
* *Paguridae*			●	●	●	-
* *Amphipoda	●	●	●	●	●	5
Mollusca						
* Mytilus edulis*	●	●	●	●	●	15
Echinodermata						
* Asterias rubens*	●	●	●	●	●	12
* Ophiothrix fragilis*	●	●		●	●	8
* Psammechinus miliaris*	●	●	●	●	●	8
Pisces**		●	●	●	●	3
Agonus cataphractus*	●			●		-
Ctenolabrus rupestris*	●			●	●	-
* *Cottidae	●					-
* *Gadidae		●	●	●	●	4
* Gadus morhua*				●		5
* *Labridae	●					4
* *Mugilidae*	●				●	-
* Trisopterus luscus*	●	●		●		4
* *Perciformes*	●			●	●	-
* *Pleuronectidae*			●	●	●	-

* Taxa not observed on GVI footage.

** Observation of unidentified Hydrozoa and Pisces.

### Species abundance estimation

Platforms P3, P4 and P5 were fully covered with marine fouling at all depths, but the composition and abundance of the marine fouling varied over depth and along the distance-from-shore gradient. Several legs on P1 and P2 up to a depth of 10 m were not fully covered. Tables 4–8 show averaged abundance estimations for the 11 high detectable species in each depth band on platforms P1 –P5, based on the Braun-Blanquet values. *Metridium senile* was the dominant species in depth range 25–45 m on all platforms, except on P4. In the depth range 0–20 m, *Mytilus edulis* was often present, especially on P1 and P2. However, it was almost completely absent from P3 and P5, platforms located further offshore. On P4 *M*. *edulis* was present up to a depth of 15 m. Rhodophyta were found on all platforms, between 0–5 m, while on P5 they were present up to 10 m. *Alcyonium digitatum* was not observed on P1 and P2, but increased along the distance-from-shore gradient at P3, P4 and P5. Porifera species were not observed on P1 and P2, but were observed on the other platforms, although in low abundance. Abundance of *Cancer pagurus*, *Asterias rubens*, *Ophiothrix fragilis*, *Necora puber* and *Psammechinus miliaris* decreased along the distance-from-shore gradient, and these species were very rare on P5.

**Table 4:** Averaged categorised abundance of the 11 high detectable taxa, with 95% confidence interval around the mean, per depth band on platform P1.

Platform P1						
Taxa	Depth band					
	0–5	5–10	10–15	15–20	20–25	25–30
Rhodophyta	3 ± 1.70	0 ± 0.00	0 ± 0.00	0 ± 0.00	0 ± 0.00	0 ± 0.00
Porifera	0 ± 0.00	0 ± 0.00	0 ± 0.00	0 ± 0.00	0 ± 0.00	0 ± 0.00
*Metridium senile*	0 ± 0.00	0 ± 0.00	6 ± 0.56	8 ± 0.40	9 ± 0.28	8 ± 0.58
Hexacorallia	4 ± 2.29	6 ± 1.11	6 ± 0.82	3 ± 1.75	2 ± 1.73	3 ± 1.81
*Alcyonium digitatum*	0 ± 0.00	0 ± 0.00	0 ± 0.00	0 ± 0.00	0 ± 0.00	0 ± 0.00
*Cancer pagurus*	0 ± 0.00	0 ± 0.58	1 ± 0.96	2 ± 0.51	2 ± 0.79	2 ± 0.79
*Necora puber*	0 ± 0.00	0 ± 0.00	0 ± 0.00	1 ± 0.84	1 ± 0.90	2 ± 0.51
*Mytilus edulis*	4 ± 2.91	6 ± 1.95	8 ± 0.51	7 ± 0.40	4 ± 1.98	0 ± 0.00
*Asterias rubens*	1 ± 1.08	3 ± 1.20	3 ± 1.43	2 ± 0.94	0 ± 0.49	1 ± 0.96
*Ophiothrix fragilis*	0 ± 0.00	2 ± 1.73	3 ± 1.54	4 ± 1.26	1 ± 1.64	0 ± 0.00
*Psammechinus miliaris*	0 ± 0.84	0 ± 0.00	1 ± 1.28	0 ± 0.00	0 ± 0.00	0 ± 0.00

The rounded abundance values are based on Table 2.

**Table 5:** Averaged categorised abundance of the 11 high detectable taxa, with 95% confidence interval around the mean, per depth band on platform P2.

Platform P2							
Taxa	Depth band						
	0–5	5–10	10–15	15–20	20–25	25–30	30–35
Rhodophyta	3 ± 3.14	0 ± 0.00	0 ± 0.00	0 ± 0.00	0 ± 0.00	0 ± 0.00	0 ± 0.00
Porifera	0 ± 0.00	0 ± 0.00	0 ± 0.00	0 ± 0.00	0 ± 0.00	0 ± 0.00	0 ± 0.00
*Metridium senile*	2 ± 1.96	3 ± 2.17	5 ± 0.49	7 ± 0.94	8 ± 0.57	9 ± 0.00	9 ± 0.00
Hexacorallia	5 ± 0.00	7 ± 0.49	7 ± 0.49	6 ± 4.00	6 ± 1.13	4 ± 2.65	0 ± 0.00
*Alcyonium digitatum*	0 ± 0.00	0 ± 0.00	0 ± 0.00	0 ± 0.00	0 ± 0.00	0 ± 0.00	0 ± 0.00
*Cancer pagurus*	0 ± 0.00	0 ± 0.49	0 ± 0.00	0 ± 0.00	0 ± 0.00	0 ± 0.49	0 ± 0.00
*Necora puber*	0 ± 0.00	0 ± 0.00	0 ± 0.00	0 ± 0.00	0 ± 0.49	0 ± 0.49	0 ± 0.57
*Mytilus edulis*	7 ± 0.49	7 ± 1.27	7 ± 0.98	0 ± 0.00	0 ± 0.00	0 ± 0.00	0 ± 0.00
*Asterias rubens*	5 ± 0.00	5 ± 0.49	4 ± 0.80	3 ± 0.94	2 ± 1.79	0 ± 0.49	0 ± 0.00
*Ophiothrix fragilis*	0 ± 0.00	0 ± 0.00	0 ± 0.00	1 ± 2.45	0 ± 0.00	0 ± 0.00	0 ± 0.00
*Psammechinus miliaris*	0 ± 0.00	0 ± 0.00	0 ± 0.00	0 ± 0.00	0 ± 0.00	0 ± 0.00	0 ± 0.00

The rounded abundance values are based on Table 2.

**Table 6:** Averaged categorised abundance of the 11 high detectable taxa, with 95% confidence interval around the mean, per depth band on platform P3.

Platform P3									
Taxa	Depth band								
	0–5	5–10	10–15	15–20	20–25	25–30	30–35	35–40	40–45
Rhodophyta	2 ± 2.32	5 ± 0.00	0 ± 0.00	0 ± 0.00	0 ± 0.00	0 ± 0.00	0 ± 0.00	0 ± 0.00	0 ± 3.54
Porifera	0 ± 0.00	0 ± 0.00	0 ± 0.00	0 ± 0.00	0 ± 0.00	0 ± 1.39	0 ± 0.00	0 ± 0.00	0 ± 0.00
*Metridium senile*	7 ± 2.02	2 ± 0.94	4 ± 1.23	5 ± 0.49	5 ± 0.57	6 ± 0.00	6 ± 0.00	6 ± 0.00	6 ± 0.00
Hexacorallia	0 ± 0.49	3 ± 1.23	4 ± 0.94	6 ± 2.93	5 ± 0.57	6 ± 1.96	3 ± 1.47	3 ± 1.96	2 ± 0.00
*Alcyonium digitatum*	4 ± 0.00	0 ± 0.00	0 ± 0.00	0 ± 0.00	0 ± 2.02	1 ± 2.26	1 ± 1.86	1 ± 0.00	2 ± 2.17
*Cancer pagurus*	2 ± 0.00	0 ± 0.00	0 ± 0.49	0 ± 0.49	0 ± 0.57	0 ± 0.49	0 ± 0.57	1 ± 0.49	0 ± 0.49
*Necora puber*	1 ± 0.49	0 ± 0.00	0 ± 0.00	0 ± 0.00	0 ± 0.49	0 ± 0.00	0 ± 0.94	1 ± 0.49	0 ± 1.39
*Mytilus edulis*	0 ± 0.00	4 ± 0.00	0 ± 0.00	0 ± 0.00	0 ± 0.00	0 ± 0.00	0 ± 0.00	0 ± 0.00	0 ± 0.00
*Asterias rubens*	0 ± 1.39	1 ± 0.00	3 ± 0.00	3 ± 0.94	2 ± 0.94	2 ± 0.00	1 ± 0.49	1 ± 0.00	0 ± 0.00
*Ophiothrix fragilis*	0 ± 0.00	0 ± 0.00	0 ± 0.00	0 ± 0.00	0 ± 0.00	0 ± 0.00	0 ± 0.00	0 ± 0.00	0 ± 0.00
*Psammechinus miliaris*	0 ± 0.00	0 ± 0.00	0 ± 0.00	0 ± 0.00	0 ± 0.00	0 ± 0.00	0 ± 0.00	0 ± 0.00	0 ± 0.00

The rounded abundance values are based on Table 2.

**Table 7:** Averaged categorised abundance of the 11 high detectable taxa, with 95% confidence interval around the mean, per depth band on platform P4.

Platform P4									
Taxa	Depth band								
	0–5	5–10	10–15	15–20	20–25	25–30	30–35	35–40	40–45
Rhodophyta	0 ± 1.42	0 ± 0.00	0 ± 0.00	0 ± 0.00	0 ± 0.00	1 ± 0.00	2 ± 0.00	1 ± 0.00	0 ± 0.00
Porifera	0 ± 0.00	0 ± 0.00	0 ± 0.00	0 ± 0.00	1 ± 0.00	1 ± 0.00	1 ± 0.98	1 ± 3.15	0 ± 0.00
*Metridium senile*	5 ± 1.11	5 ± 0.94	6 ± 1.21	7 ± 1.04	6 ± 0.82	4 ± 1.34	2 ± 1.28	5 ± 0.63	5 ± 1.44
Hexacorallia	5 ± 0.00	5 ± 1.55	2 ± 1.04	1 ± 1.89	1 ± 2.10	3 ± 2.03	5 ± 1.83	6 ± 0.98	4 ± 0.00
*Alcyonium digitatum*	1 ± 0.00	1 ± 0.00	1 ± 0.00	3 ± 0.00	5 ± 0.00	4 ± 1.55	2 ± 1.96	3 ± 1.65	2 ± 0.48
*Cancer pagurus*	0 ± 0.00	0 ± 0.00	0 ± 0.47	0 ± 0.22	1 ± 0.58	0 ± 0.48	0 ± 0.66	0 ± 0.67	0 ± 0.39
*Necora puber*	0 ± 0.24	0 ± 0.24	0 ± 0.24	0 ± 0.24	0 ± 0.00	0 ± 0.24	0 ± 0.32	0 ± 0.00	0 ± 0.00
*Mytilus edulis*	0 ± 0.36	0 ± 2.16	0 ± 1.71	0 ± 0.00	0 ± 0.00	2 ± 0.00	2 ± 0.00	2 ± 0.00	1 ± 0.00
*Asterias rubens*	2 ± 0.52	2 ± 0.86	2 ± 0.24	1 ± 0.73	1 ± 0.36	1 ± 0.90	0 ± 0.91	1 ± 0.73	1 ± 0.39
*Ophiothrix fragilis*	0 ± 0.00	0 ± 0.00	0 ± 0.00	0 ± 0.00	0 ± 0.00	0 ± 0.00	0 ± 0.00	0 ± 0.00	0 ± 0.00
*Psammechinus miliaris*	0 ± 0.84	0 ± 0.00	0 ± 1.28	0 ± 0.00	0 ± 0.00	0 ± 0.00	0 ± 0.00	0 ± 0.00	0 ± 0.00

The rounded abundance values are based on Table 2.

**Table 8:** Averaged categorised abundance of the 11 high detectable taxa, with 95% confidence interval around the mean, per depth band on platform P5.

	Platform P5							
Taxa	Depth band							
	0–5	5–10	10–15	15–20	20–25	25–30	30–35	35–40
Rhodophyta	4 ± 1.90	1 ± 1.44	0 ± 0.00	0 ± 0.00	0 ± 0.00	0 ± 0.00	0 ± 0.00	0 ± 0.00
Porifera	0 ± 0.00	1 ± 1.57	2 ± 1.92	0 ± 0.00	0 ± 0.00	0 ± 0.00	0 ± 0.00	0 ± 0.00
*Metridium senile*	0 ± 0.78	5 ± 0.78	6 ± 0.78	8 ± 0.48	9 ± 0.00	9 ± 0.00	9 ± 0.00	9 ± 0.39
Hexacorallia	9 ± 0.00	8 ± 0.48	8 ± 0.39	7 ± 0.62	4 ± 1.96	2 ± 1.92	0 ± 0.00	4 ± 1.82
*Alcyonium digitatum*	0 ± 0.00	5 ± 0.00	5 ± 0.39	3 ± 2.74	3 ± 2.40	1 ± 1.57	3 ± 2.11	5 ± 0.00
*Cancer pagurus*	0 ± 0.00	0 ± 0.00	0 ± 0.00	0 ± 0.00	0 ± 0.00	0 ± 0.00	0 ± 0.39	0 ± 0.00
*Necora puber*	0 ±±0.00	0 ± 0.00	0 ± 0.00	0 ± 0.00	0 ± 0.00	0 ± 0.00	0 ± 0.00	0 ± 0.00
*Mytilus edulis*	0 ± 0.78	0 ± 0.00	0 ± 0.00	0 ± 0.00	0 ± 0.00	0 ± 0.00	0 ± 0.00	0 ± 0.00
*Asterias rubens*	0 ± 0.00	0 ± 0.00	0 ± 0.00	0 ± 0.00	0 ± 0.00	0 ± 0.00	0 ± 0.00	0 ± 0.00
*Ophiothrix fragilis*	0 ± 0.00	0 ± 0.00	0 ± 0.00	0 ± 0.00	0 ± 0.00	0 ± 0.00	0 ± 0.00	0 ± 0.00
*Psammechinus miliaris*	0 ± 0.00	0 ± 0.00	0 ± 0.00	0 ± 0.00	0 ± 0.00	0 ± 0.00	0 ± 0.00	0 ± 0.00

The rounded abundance values are based on Table 2.

**Fig 2:**
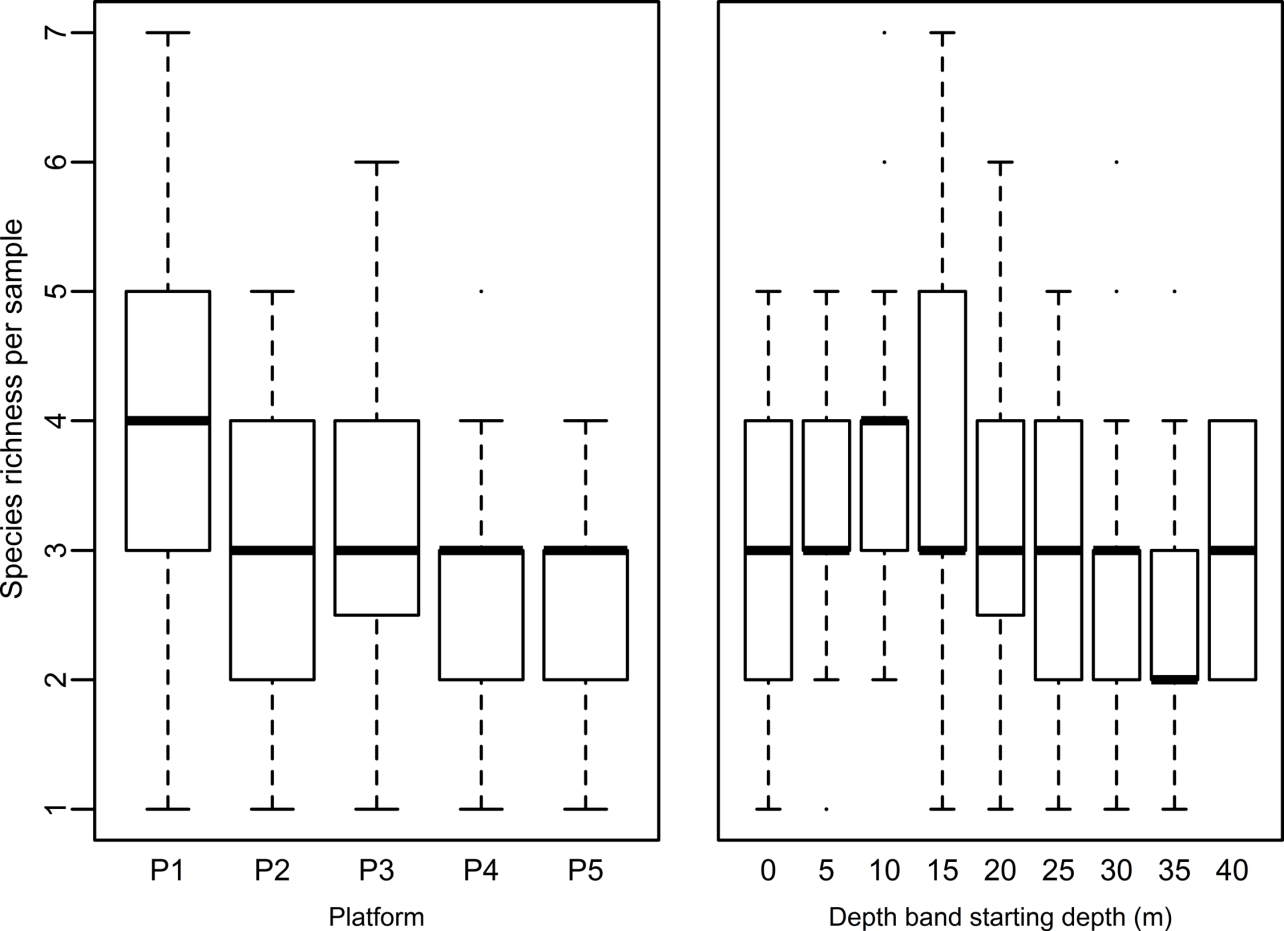
Species richness per platform & depth band. Boxplot showing the number of species per sample (n = 215), per platform with all depths combined (left image) and per depth-band with all platforms (n = 5) combined.

**Fig 3:**
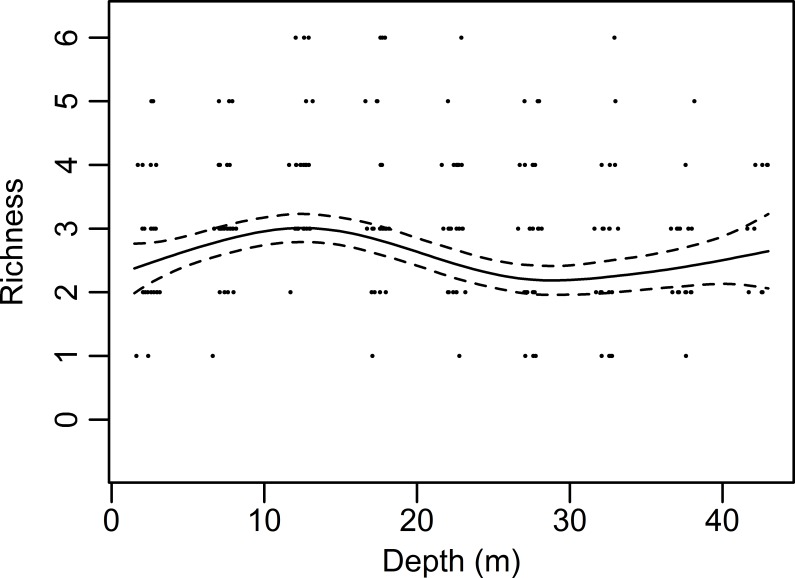
Modelled relation of species richness (S) with depth. Plot of the fitted Generalised Additive Model smoother showing the significant non-linear effect of depth (p = 0.001) on the species richness (S) on all platforms.

The observed species richness (S) categorised in depth bands and platforms is shown in Fig 2. Model selection for the GAM resulted in the inclusion of depth, the interaction between distance from shore and the community age and video length as explanatory variables, which explained 42% of the deviance. S increased significantly with increasing community age and video length (p<0.001). With increasing distance from shore, S decreased significantly although this effect interacted with the age of the community (p<0.001). Depth showed a non-linear significant relationship with species richness (p = 0.001). Species richness initially increased with depth, but then decreased again after 15–20 m (Fig 3).

PERMANOVA showed that depth, community age, quality of the footage and the interaction effect between distance and community age have a significant effect (p≤0.001) on the species assemblages found on offshore platforms (Table 9). Total video length varied per platform, but PERMANOVA showed this had no significant effect.

**Table 9:** PERMANOVA on variables that influence species assemblages significantly.

Source	d.f.	SS	MS	F	R^2^	P
Depth	1	11.031	11.0311	113.085	0.28374	0.001
Age	1	2.518	2.5179	25.812	0.06477	0.001
Quality	1	1.080	1.0801	11.073	0.02778	0.001
Age:Distance	1	3.763	3.7629	38.576	0.09679	0.001
Residuals	210	20.485	0.0975		0.52692	
Total	214	38.877			1.00000	

D.f. = degrees of freedom. SS = sum of squares. MS = mean of squares. Age:Distance tests the effect of the interaction between these two variables. Age refers to the age of the community.

## Discussion

The present study provides insight in the composition of the species assemblages found on five offshore gas platforms in the southern North Sea. Our results show that a variety of marine species are found on these offshore gas platforms. Many thousands of artificial reef structures are present in the North Sea in the form of shipwrecks, wind farms and oil & gas platforms [71,72]. Furthermore, thousands of wind turbine foundations will be installed in the North Sea in the near future [26]. Before the onset of industrial fisheries, large areas of the southern North Sea bottom were covered with natural reefs, many of which are now lost [60,73]. Artificial reefs in this area might compensate for the loss of this habitat. In previous studies, 90% of the species present on artificial hard substrata in the southern North Sea were shown to be absent in the soft bottomed surroundings [74]. The presence of an artificial object will therefore have a very strong effect on the local biodiversity, almost doubling it [75]. However, it remains unclear to what extent the species assemblages present on artificial reefs resemble those of natural reefs. Published species observations as presented in the current study and many others, are needed to evaluate the ecological value of current and future artificial reefs.

### Trends in community composition

Although similarities in species assemblages are found between offshore platforms, there are also striking differences. Especially the abundance of *Mytilus edulis* on P4 differs from the other far offshore platforms, P3 and P5. With 3 years community age at all depths, P4 is young compared to the minimum age of 7 and 13 years for P3 and P5, respectively. This suggests that *M*. *edulis* is an early colonizer of offshore platforms, which is confirmed by wind farm colonisation studies in the southern North Sea where *M*. *edulis* was dominant in the first years after construction [8,49]. *M*. *edulis* growth rate is dependent on food availability [76], explaining the high abundance in the depth range 0–20 m on platforms closer to shore, where food concentrations are higher [77].

*Alcyonium digitatum* was not observed on P1 and P2, but was observed on the other platforms. Abundance of *A*. *digitatum* correlated positively with distance from shore, in line with the pattern found on ship wrecks in the Belgian part of the North Sea, where *A*. *digitatum* was only found on wrecks far offshore and never close to shore [78]. This may be explained by a water temperature or food availability gradient, since both decrease with distance from shore in the southern North Sea [77].

Species with a low detectability score were omitted from the data. Using these adjusted data in the GAM, the species richness was highest on P1 and decreased with increasing distance from shore. However, this effect interacted with the community ages, which varied between 3 and 39 years. A similar significant interaction was found in the PERMANOVA results. Platforms are cleaned regularly between the water surface and approximately 10 m water depth, depending on the abundance of hard marine growth (e.g. mussels and barnacles). Platforms closer to shore were cleaned more recently than those further offshore. Cleaning effectively resets community succession every few years, keeping it in a continuous young stage, affecting the species composition. This may explain the significant effect of the age:distance interaction, obscuring the distance from shore effect communicated by other authors [6,7]. The operator of the platforms informed us that platforms close to shore indeed are cleaned more often than locations far offshore.

The lower richness in shallow parts, as shown by the GAM, can also be explained by the impact of higher wave action near the surface, which is known to decrease species richness [79]. In the deeper parts of the platform the richness was also significantly lower than at intermediate depths, caused by the dominance of a limited amount of taxa. Anemones such as *Metridium senile* are known to deter other organisms [80], explaining the lower species richness around these species. A similar effect was observed on rocky reefs in the Netherlands [60]. Both the wave disturbance and deterring effect of *M*. *senile* are in line with the intermediate disturbance hypothesis, which states that biodiversity is highest at intermediate disturbance rates and smaller at high and low rates [81]. It is suggested that at low rates of disturbance, strong competitors exclude competitively inferior species, whereas at high rates of disturbance, recruitment cannot balance the high rates of mortality, and slow recruiting species disappear from the community. This effect was most prominent on P5, where the deeper parts of the platform were dominated by *Metridium senile* and *Alcyonium digitatum*.

Our results are in line with research on offshore platforms in other waters, where depth was also found to have a significant influence on species composition of the marine fouling [6,7]. However, depth, community age, quality of the footage and the age:distance from shore interaction only explained 47% of the variance in the PERMANOVA and 42% of the deviance in the GAM. The amount of unexplained variation indicates that other environmental variables, such as salinity, water temperature, water currents, food supply, light penetration, silt content and the position on the leg (interior/exterior) in relation to the direction of the current may also play a role [2,82].

### Evaluation of ROV footage used for species identification

Data used in this study were collected from images collected for technical inspection. The use of such images is a time and cost effective method to gain insight in the organisms present on offshore platforms. It allows for the inventory of large species present on vast amounts of surface area, in all depths, which can be challenging using other methods such as diving surveys [60]. Many locations can be investigated and if needed, several years are available for time series analysis [3]. Furthermore, identifications are easily confirmed by peers, increasing the quality of the data.

However, ROV inspection footage is created to obtain an overview of the technical integrity of the installation, not for biological study. As such, the quality was often insufficient to identify taxa to species level. Furthermore, video footage will only show the organisms on top of the fouling layer, missing species in the deeper layers. Therefore, the number of taxa identified in this study is an underestimation of the true number of species present. For a thorough overview of the species present, a combination of methods such as destructive sampling for small organisms and *in situ* observations for rare, fast moving or inconspicuous organisms should be applied, as shown on rocky reefs in the southern North Sea [60].

### Conclusion

Using ROV footage, a total of 30 taxa were identified in the species assemblages on five offshore gas platforms in the southern North Sea. Species richness initially increased with depth, but decreased after 15–20 m. Species richness decreased significantly with increasing distance from shore; although, this effect may be obscured by the younger community age in <10 m depths on platforms closer to shore resulting from the regular cleaning of these platforms. Not all variability was explained by depth and the distance from shore effect, indicating that other environmental variables also play a role. Further research with higher quality images, *in situ* observations and sampling of the marine fouling is needed to understand what other environmental variables influence the species assemblages on offshore platforms.
